# The Global Network Socioeconomic Status Index as a predictor of stillbirths, perinatal mortality, and neonatal mortality in rural communities in low and lower middle income country sites of the Global Network for Women’s and Children’s Health Research

**DOI:** 10.1371/journal.pone.0272712

**Published:** 2022-08-16

**Authors:** Archana B. Patel, Carla M. Bann, Cherryl S. Kolhe, Adrien Lokangaka, Antoinette Tshefu, Melissa Bauserman, Lester Figueroa, Nancy F. Krebs, Fabian Esamai, Sherri Bucher, Sarah Saleem, Robert L. Goldenberg, Elwyn Chomba, Waldemar A. Carlo, Shivaprasad Goudar, Richard J. Derman, Marion Koso-Thomas, Elizabeth M. McClure, Patricia L. Hibberd

**Affiliations:** 1 Lata Medical Research Foundation, Nagpur, India; 2 Datta Meghe Institute of Medical Sciences, Wardha, India; 3 RTI International, Research Triangle Park, NC, United States of America; 4 Kinshasa School of Public Health, University of Kinshasa, Kinshasa, Democratic Republic of Congo; 5 University of North Carolina at Chapel Hill, Chapel Hill, NC, United States of America; 6 Instituto de Nutrición de Centroamérica y Panamá, Guatemala City, Guatemala; 7 University of Colorado School of Medicine, Denver, CO, United States of America; 8 Moi University School of Medicine, Eldoret, Kenya; 9 Indiana School of Medicine, University of Indiana, Indianapolis, IN, United States of America; 10 Aga Khan University, Karachi, Pakistan; 11 Columbia University School of Medicine, New York, NY, United States of America; 12 University Teaching Hospital, Lusaka, Zambia; 13 University of Alabama at Birmingham, Birmingham, AL, United States of America; 14 KLE Academy Higher Education and Research, J N Medical College Belagavi, Karnataka, India; 15 Thomas Jefferson University, Philadelphia, PA, United States of America; 16 Eunice Kennedy Shriver National Institute of Child Health and Human Development, Bethesda, MD, United States of America; 17 Department of Global Health, Boston University School of Public Health, Boston, MA, United States of America; University of Lausanne: Universite de Lausanne, SWITZERLAND

## Abstract

**Background:**

Globally, socioeconomic status (SES) is an important health determinant across a range of health conditions and diseases. However, measuring SES within low- and middle-income countries (LMICs) can be particularly challenging given the variation and diversity of LMIC populations.

**Objective:**

The current study investigates whether maternal SES as assessed by the newly developed Global Network-SES Index is associated with pregnancy outcomes (stillbirths, perinatal mortality, and neonatal mortality) in six LMICs: Democratic Republic of the Congo, Guatemala, India, Kenya, Pakistan, and Zambia.

**Methods:**

The analysis included data from 87,923 women enrolled in the Maternal and Newborn Health Registry of the NICHD-funded Global Network for Women’s and Children’s Health Research. Generalized estimating equations models were computed for each outcome by SES level (high, moderate, or low) and controlling for site, maternal age, parity, years of schooling, body mass index, and facility birth, including sampling cluster as a random effect.

**Results:**

Women with low SES had significantly higher risks for stillbirth (p < 0.001), perinatal mortality (p = 0.001), and neonatal mortality (p = 0.005) than women with high SES. In addition, those with moderate SES had significantly higher risks of stillbirth (p = 0.003) and perinatal mortality (p = 0.008) in comparison to those with high SES.

**Conclusion:**

The SES categories were associated with pregnancy outcomes, supporting the validity of the index as a non–income-based measure of SES for use in studies of pregnancy outcomes in LMICs.

## Introduction

Income inequality has been on the rise globally. The U.N. Sustainable Development Goal 10 is solely dedicated to reducing this inequality within and among countries. Globally, socioeconomic status (SES) is an important health determinant across a range of health conditions and diseases and plays a major role in maternal and child health outcomes [1]. SES of the household is a direct enabler to obtain quality health care because it allows better access and affordability when a higher level of care is needed. SES also indirectly impacts health; higher level of education, better access to clean water and sanitation, improved nutrition, and awareness of healthy practices all reduce risk of household illnesses [2]. Thus, it has been observed that the lower the SES of an individual, the worse their health status, secondary to the association of low SES with reduced health-seeking behavior and limited options for accessibility and affordability of good health care.

Economies in low- and middle-income countries (LMICs) are often fluid, informal, and undocumented [3]. Identifying a common robust and reliable method to assess SES across different LMIC sites in a multisite study is particularly challenging given the variation and diversity of the populations [4]. The method needs to reliably capture SES characteristics that are likely to impact health outcomes both within and across sites. An SES index should be able to economically position individuals in relation to other members of the community. In socialist countries that make quality health care available and affordable to even those from lower SES, the disparity in health outcomes among the high and low SES may not be easy to quantify [5]. Thus, there can be varying impacts of SES within and across countries. It is especially challenging to assess SES in LMICs and the extent of its impact on health outcomes [6]. Hence, it is necessary to assess the impact of SES on health outcomes because SES may confound the impact of interventions. SES assessment also helps to prioritize public health actions to those who need it most.

Since 2009, the *Eunice Kennedy Shriver* National Institute of Child Health and Human Development’s (NICHD’s) Global Network (GN) for Women’s and Children’s Health Research has supported a population-based Maternal and Newborn Health Registry (MNHR) of pregnant women and their babies living in seven rural and semi-urban study sites across three Asian, three sub-Saharan African, and one Central American country. In 2016, a questionnaire was added to collect data on items adapted from the Multidimensional Poverty Index (MPI) assessed to be relevant to the study sites [7]. A brief index, the GN-SES Index (GN-SESI) demonstrated good internal consistency and reliability [8]. The scores were significantly associated with formal education, years of education, having received antenatal care, and facility delivery [8]. Given prior research demonstrating a relationship of SES status with health outcomes, we hypothesized that women with higher scores on the GN-SESI would have better pregnancy outcomes. The objective of the current research is to evaluate the association of the GN-SESI with MNHR pregnancy outcomes including occurrence of stillbirths, perinatal mortality, and neonatal mortality in women enrolled in the MNHR.

## Methods

### Study design and setting

NICHD’s GN is a multisite research network that represents partnerships between U.S. and international investigators. The GN-MNHR has been collecting prospective data on a population-based sample of pregnant women and their babies since 2009. The details of the MNHR have been previously published [9]. Since its inception, the MNHR has registered more than 750,000 pregnant women and their babies in rural and semi-urban communities in the following countries by region: Africa (Democratic Republic of Congo [DRC], Kenya, and Zambia), Asia (Belagavi [India], Nagpur [India], and Pakistan), and Central America (Guatemala). Each site comprises between 6 and 24 distinct geographic locations (clusters) [9].

Throughout the study period, pregnant women within the defined geographic catchment areas were recruited as early as possible during pregnancy (baseline assessment) and followed through labor and delivery (birth assessment) to 42 days postpartum (outcome assessment) to obtain maternal, fetal, and neonatal outcomes. Written informed consent was obtained for enrollment in the study. For participants who were minors, verbal assent was obtained from the minor and a written signature was obtained from a family member providing permission for the minor to participate in the study.

The MNHR began collecting data on SES indicators, including items on living conditions and household assets at all sites, in 2016. Specific training materials were developed for administration of the SES questions, and all study data were subject to the GN’s standard quality control procedures for outcome ascertainment [9].

### Ethical approvals

The MNHR study and questions used to devise SES were reviewed and approved by all institutions’ ethics review committees at each recruiting site and all U.S.-based partner institutions. The study was registered at ClinicalTrials.gov (NCT01073475). A Data Safety Monitoring Committee appointed by NICHD reviewed the study data on an annual basis.

### Study participants

All women enrolled in MNHR were asked to complete the GN-SES Index questionnaire starting in 2016. For this analysis, participants were included only if they had SES index scores. Women who experienced miscarriage or medical termination of pregnancy were excluded from analyses.

### Global Network SES index

The GN-SESI includes the following 12 items adapted from the MPI on housing conditions and assets owned by the participant’s household: finished floor material, flush toilet, Liquid Petroleum Gas/electricity for cooking fuel, improved source of drinking water, more than one room in the home, electricity in home, television, refrigerator, smart phone, car, motorbike, and bicycle [7]. Scores range from 0 to 100. Item response theory scoring was used to allow for the inclusion of site-specific items while maintaining a common set of core items across the sites, resulting in comparable scores that also account for site-level variability in indicators of wealth [9]. We described the details of the index development and validation process in a previous paper [8] where the index demonstrated good internal consistency (alpha = 0.81) and construct validity. For this analysis, we classified participants into three groups based on index scores with each category representing one-third of the possible score range: 1) Low SES (score < 33), 2) Moderate SES (score 33–66), and 3) High SES (score ≥ 66). While similar results were found when analyzing SES scores as a continuous variable, we divided scores into these three categories to facilitate interpretation of results.

### Outcome definitions

Stillbirths were defined as birth of a baby after 20 weeks/500 g that showed no signs of life at birth (i.e., no gasping, breathing, heartbeat, or movement), regardless of fresh or macerated appearance. Perinatal mortality was defined as a stillbirth or neonatal death at less than 7 days. Neonatal mortality was defined as the death of a live-born baby before 28 days.

### Statistical analysis

We compared demographic characteristics across the three levels (Low, Moderate, and High SES) using chi-square tests for categorical variables and analyses of variance for continuous variables. Cochran-Armitage trend tests were used to test for a linear trend in the proportions of participants experiencing each outcome (stillbirth, perinatal mortality, neonatal mortality) across the three SES levels. These proportions were converted to rates per 1,000 to allow for comparisons with other sources of data regarding country-level outcomes.

To further examine the relationship between SES index and outcomes, we fit generalized estimating equations models for each outcome by SES level, controlling for site, maternal age, parity, years of schooling, WHO categories of body mass index (BMI) [10], and facility birth, and including cluster as a random effect to account for nesting by cluster. We tested different specifications for the working correlation structure for the models and selected the correlation structure that produced the lowest QIC statistic [11].

Based on these models, we calculated adjusted relative risks and 95% confidence intervals comparing risks of poor outcomes across SES levels. We applied a Benjamini-Hochberg adjustment to control for Type 1 errors of the pairwise comparisons for each outcome. Analyses were run for the overall sample and each site individually.

## Results

**Fig 1:**
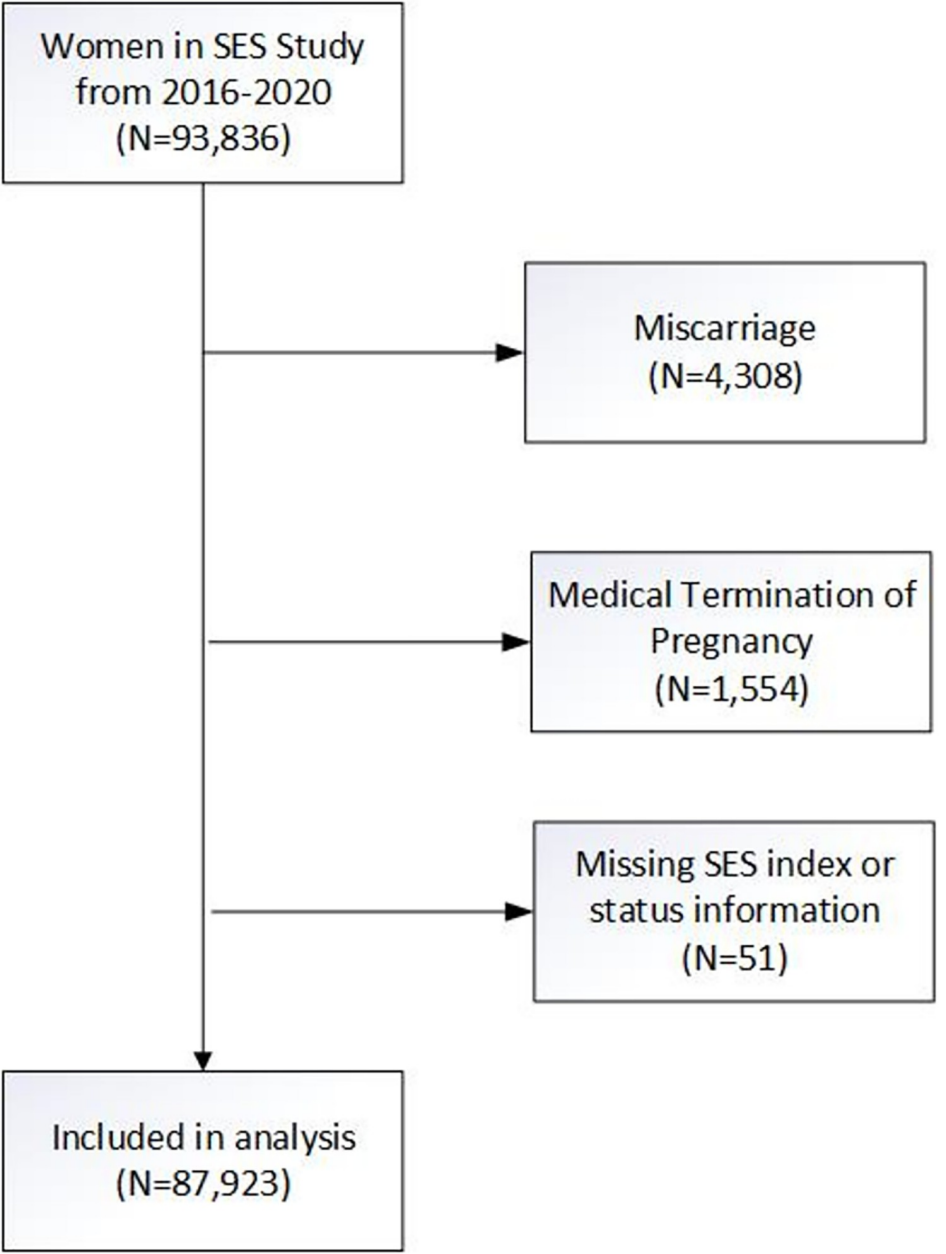
CONSORT diagram of participant flow.

A total of 94.435 women were consented for study participation and enrolled in the study and were administered the SES questions from February 2016 to February 2020 (Fig 1). Women with miscarriage or medical termination of pregnancy or who were missing data on the GN-SESI or delivery outcomes were excluded, resulting in a final sample size of 87,923 (93.1%). Of this sample, all 87,923 had data on stillbirth outcome, 87,773 had data on perinatal mortality, and 85,659 had data on neonatal mortality.

Across all sites, 38,373 (44%) women had Low SES, 28,448 (32%) Moderate SES, and 21,102 (24%) High SES. Demographic characteristics by SES level are shown in Table 1. Mean (SD) of maternal age in years varied significantly across the three SES levels with p < 0.001: Low (25.45 (6.33)), Moderate (25.06 (5.58)), and High (24.71 (4.66)) (not shown). There were significant differences across the SES levels for all the characteristics (p < 0.001) (Table 1). Those with High SES had lower parity, more years of formal schooling, and greater likelihood of delivering at a facility. Obesity rates were lower in the Low SES group. The distribution of participants by site varied across the three SES categories. The Low SES group included more participants from the African sites (DRC, Kenya, and Zambia) while the High SES group included more participants from the Asian sites (India and Pakistan) and Guatemala.

**Table 1:** Sample demographic characteristics by Socioeconomic Status (SES).

	All (N = 87,923)	Low (N = 38,373)	Moderate (N = 28,448)	High (N = 21,102)	p-value
Characteristic	N (%)	N (%)	N (%)	N (%)	
**Parity**					
0	29,125 (33)	10,135 (26)	9,611 (34)	9,379 (44)	< 0.001
1–2	36,388 (41)	13,802 (36)	12,762 (45)	9,824 (47)	
3+	22,383 (25)	14,413 (38)	6,072 (21)	1,898 (9)	
**Years of formal schooling**					
0	15,717 (18)	10,370 (27)	3,988 (14)	1,359 (6)	< 0.001
1–6	18,514 (21)	10,152 (26)	6,216 (22)	2,146 (10)	
7–12	46,909 (53)	16,912 (44)	16,755 (59)	13,242 (63)	
> 12	6,763 (8)	930 (2)	1,485 (5)	4,348 (21)	
**Facility birth**	72,088 (82)	29,430 (77)	23,326 (82)	19,332 (92)	< 0.001
**BMI**					
Underweight	13,749 (16)	4,149 (11)	4,903 (17)	4,697 (22)	< 0.001
Normal	53,039 (61)	26,809 (71)	15,201 (54)	11,029 (52)	
Overweight	15,833 (18)	5,970 (16)	6,105 (22)	3,758 (18)	
Obese	4,882 (6)	1,082 (3)	2,192 (8)	1,608 (8)	
**Site**					
DRC	11,943 (14)	11,585 (30)	356 (1)	2 (0)	< 0.001
Guatemala	16,760 (19)	3,096 (8)	8,503 (30)	5,161 (24)	
India (Belagavi)	11,650 (13)	1,055 (3)	5,145 (18)	5,450 (26)	
India (Nagpur)	13,675 (16)	1,126 (3)	4,875 (17)	7,674 (36)	
Kenya	13,662 (16)	11,171 (29)	2,105 (7)	386 (2)	
Pakistan	8,602 (10)	4,495 (12)	2,539 (9)	1,568 (7)	
Zambia	11,631 (13)	5,845 (15)	4,925 (17)	861 (4)	

Note: SES scores are categorized into levels as follows: Low (0–32), Moderate (33–66), and High (67–100). p-value is based on a chi-square test comparing demographics across SES levels.

Unadjusted percentages of participants with each outcome by site and SES level are shown in Table 2. Higher rates of stillbirths and perinatal and neonatal mortality were observed across all GN-SESI categories in Pakistan. Generally, higher SES was associated with lower rates of all adverse outcomes among Guatemala and the Asian sites. Significant trends for decreasing percentages of stillbirths with increasing levels of SES were found for Guatemala (p < 0.001), Belagavi, India (p = 0.021), Nagpur, India (p = 0.016), and Pakistan (p = 0.005). In addition, higher SES was associated with lower percentages for perinatal mortality in Guatemala (p < 0.001), Belagavi, India (p = 0.009), and Nagpur, India (p = 0.002). A similar relationship was found for SES and neonatal mortality in Guatemala (p < 0.001) and Nagpur, India (p = 0.020).

**Table 2:** Mortality rates per 1,000 by site and SES.

				Linear Trend Test	
Outcome	Low	Moderate	High	Z-statistic	p-value
**Stillbirth**					
Democratic Republic of the Congo	37.3	37.9	—	—	—
Guatemala	24.1	18.2	11.2	4.41	< 0.001
India (Belagavi)	38.4	22.5	21.7	2.31	0.021
India (Nagpur)	27.4	18.8	16.2	2.41	0.016
Kenya	17.9	22.8	—	—	—
Pakistan	52.0	50.5	30.9	2.83	0.005
Zambia	18.8	17.6	21.4	-0.05	0.963
**Perinatal mortality (Stillbirth or neonatal mortality < 7 days)**					
Democratic Republic of the Congo	57.2	72.3	—	—	—
Guatemala	44.6	33.6	22.4	5.36	< 0.001
India (Belagavi)	62.4	39.6	37.9	2.62	0.009
India (Nagpur)	44.5	35.5	28.4	3.09	0.002
Kenya	29.7	31.6	—	—	—
Pakistan	98.9	93.1	81.4	1.76	0.078
Zambia	28.4	24.4	30.0	0.55	0.589
**Neonatal mortality (< 28 days)**					
Democratic Republic of the Congo	21.1	33.1	—	—	—
Guatemala	33.2	22.8	13.9	5.58	< 0.001
India (Belagavi)	28.3	20.7	18.9	1.65	0.100
India (Nagpur)	24.3	19.4	15.5	2.32	0.020
Kenya	13.5	9.4	—	—	—
Pakistan	55.7	54.1	59.2	-0.34	0.736
Zambia	11.8	8.4	12.1	1.00	0.316

Note: P-value is based on a two-sided Cochran-Armitage trend test of the proportions of participants experiencing each outcome. Proportions were converted to mortality rates per 1,000 to allow for comparisons with other sources of country-level statistics. Mortality rate was not estimated for high SES for the Democratic Republic of the Congo and Kenya due to the small number of participants (< 5%) with high SES at these sites.

**Fig 2:**
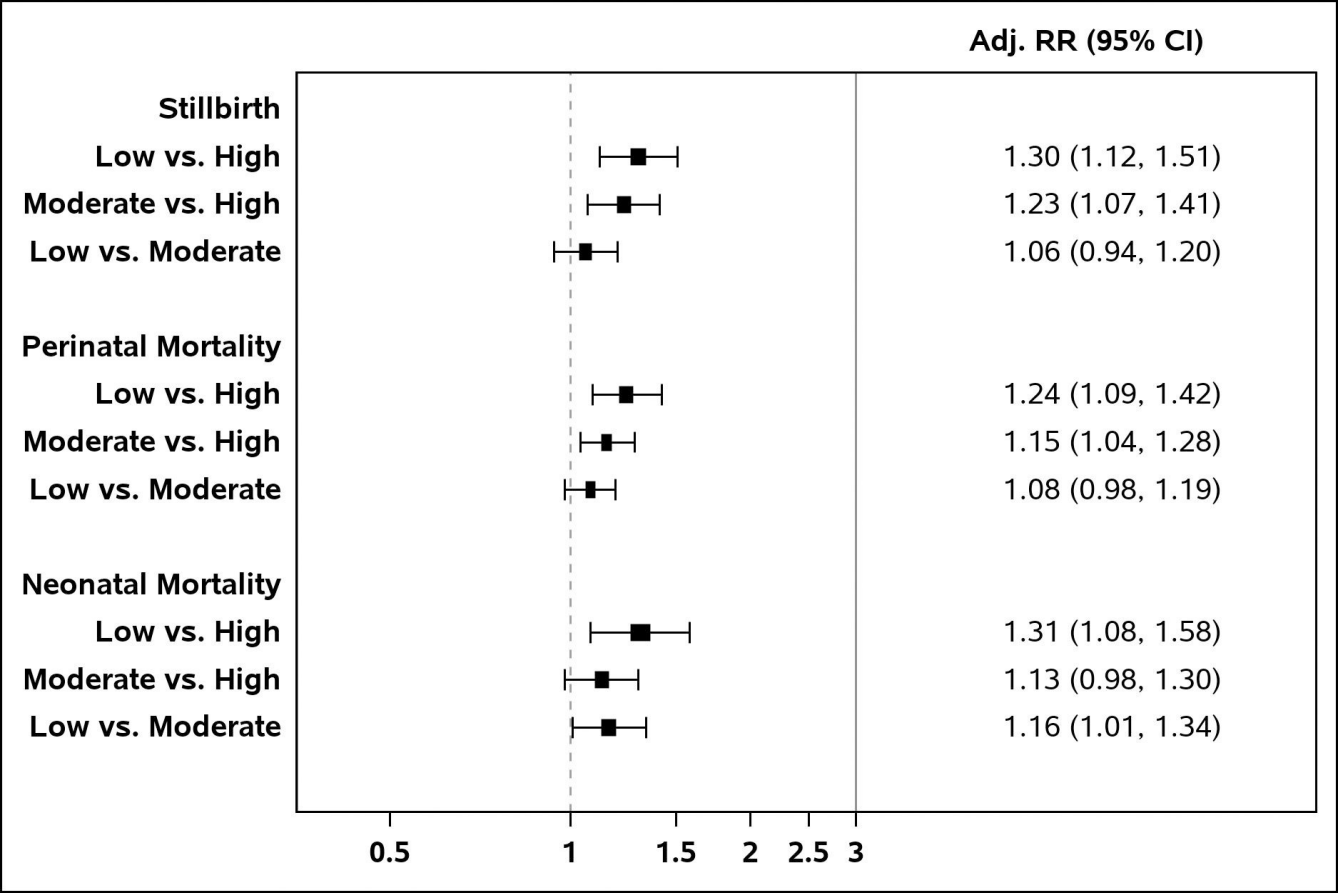
Adjusted relative risks (95% confidence intervals) of outcomes by SES: All sites. Relative risks are adjusted for SES category, site, maternal age, parity, formal education level, BMI category, and facility birth.

Adjusted relative risks of outcomes by SES level are shown in Fig 2. Higher risks of stillbirth were found among those with Low SES (RR (95% CI) = 1.30 (1.12, 1.51), p < 0.001) and Moderate SES (RR (95% CI) = 1.23 (1.07, 1.41), p = 0.003) compared to those with High SES. A similar pattern was found for perinatal mortality. Risks for perinatal mortality were higher among those with Low SES (RR (95% CI) = 1.24 (1.09, 1.42), p = 0.001) and Moderate SES (RR (95% CI) = 1.15 (1.04, 1.28), p = 0.008) in comparison to those with High SES. In addition, risks of neonatal mortality were significantly higher for those with Low versus High SES (RR (95% CI) = 1.31 (1.08, 1.58), p = 0.005) and with Low versus Moderate SES (RR (95% CI) = 1.16 (1.01, 1.34), p = 0.039). Site-specific analyses are shown in S1–S3 Figs.

## Discussion

This study found that SES Index levels were associated with pregnancy outcomes among women enrolled in the MNHR. Overall, women in the High SES category had distinctly better pregnancy outcomes with lower rates of stillbirths, perinatal mortality, and neonatal mortality than those in the Low and Moderate SES categories. All outcomes of stillbirth, perinatal mortality, and neonatal mortality were similar in the Low and Moderate SES categories when adjusted for site, maternal age, parity, years of schooling, BMI, and facility birth.

Demographic indicators such as mother’s age, years of education, family size, and family economic status are common determinants of access to health care and consequently health outcomes [12, 13]. Economic status evaluated by using family income is unreliable because of poor reporting and non-availability of an authentic database of household income [14]. Therefore, in multisite epidemiological and intervention studies in LMICs an alternate measure of determining economic status of the family to assess its impact on health outcomes within and across sites was needed [15–17].

The index was able to demographically distinguish those in High SES from those in Low or Moderate SES. Participants from the High SES categories had more years of education and lower parity. Higher SES of the GN-SESI was associated with higher education, increased frequency of antenatal visits, and increased facility births was also demonstrated previously [8]. In our earlier study we demonstrated that for facility births, fewer antenatal visits and lower levels of literacy were associated with lower levels of SES [9]. SES could indicate access to, and health care utilization, which are dependent on literacy and quality of available health care. However, women with High SES, who form a quarter of the population, may have lower rates of literacy and anemia, higher parity and poor access and utilization of health care, and suboptimal quality of care, which may have resulted in adverse neonatal and perinatal outcomes. Kenya and Zambia had more participants from the Low SES category. The lack of trend in neonatal outcomes in Zambia could be because despite the SES of a population, outcomes are dependent on availability and quality of care, which is suboptimal in LMICs. This explanation can also be used to justify the lack of trend in Pakistan for its neonatal outcomes. Among the Asian sites, Pakistan’s study population consisted mainly of High SES with few from the Low SES category. This suggests that even though households in Pakistan may have resources and asset ownership, this may not be reflected in their health outcomes [18]. Perinatal mortality was among the highest when compared to other GN sites. This could perhaps be because of very low quality of medical care in many institutions in Pakistan, low literacy status, which influences hygiene and sanitation, use of alternative traditional forms of treatment that may not be effective, inadequate maternal nutrition, and poor health care–seeking behavior [19–21].

SES index was a reliable proxy for both income and quality of health care. Higher per capita income is associated with better health outcomes and higher life expectancy at birth, and the GN-SESI was able to validate this hypothesis. Our observations are also consistent with the demographic health survey data of these countries despite the index being parsimonious and pragmatic [22, 23]. However, Pakistan is an exception; the percentage of women in the High SES category was not insignificant was comparatively lower when compared to other GN sites. For DRC, the population was uniformly poor compared to other sites and had no participants in the High SES category. The health care systems in DRC are suboptimal so the perinatal outcomes in both the Low and Moderate SES categories were similar.

The strengths of the GN-SESI index are the following: It was developed from a population-based registry at seven sites in South Asia, sub-Saharan Africa, and Central America using item response theory parameters that ensured that the SES items selected using consensual approach of site investigators worked well across the range of SES present at these sites. The index obtained information on household assets, and the SES categories were found to be reliable and verifiable when compared to demographics known to be associated with those in different SES levels [24]. Furthermore, the index categories were differently associated with pregnancy outcomes. High SES women had distinctly better outcomes than Moderate and Low SES women where the outcomes tended to be similar. This index would therefore serve as a reliable indicator that shows an association with participant outcomes. National SES indices may not be applicable across countries or for a particular site within a country and so may have limited association with the site population and its health outcomes. The index successfully addressed this gap and illustrated the need for developing study-specific indices of economic status.

The limitations of the GN-SESI index are the following: Although it is relevant to the region where the study was conducted, the GN-SESI metrics may not be representative of the country in which our GN sites were based. Middle and Low SES were not as distinctly different in their outcomes compared to High SES. These SES categorizations may not be comparable with national data that use other measures of SES or to other study settings that may use other methods to ascertain SES. Its applicability beyond the GN study sites needs to be assessed. Also, in some sites such as Kenya, the available data were sparse.

In conclusion, the GN-SESI’s ratings of the economic status of sites were consistent with the economic condition of those countries [8]. This asset class index was a reliable proxy for income and quality health care as favorable pregnancy outcomes were observed with the High SES category without asking intimidating questions about income.

## Supporting information

S1 Fig Adjusted relative risks (95% confidence intervals) of stillbirth by site and SES. Relative risks are adjusted for SES category, site, site by SES interaction, maternal age, parity, formal education level, BMI category, and facility birth.
(TIF)

S2 Fig Adjusted relative risks (95% confidence intervals) of perinatal mortality by site and SES. Relative risks are adjusted for SES category, site, site by SES interaction, maternal age, parity, formal education level, BMI category, and facility birth.
(TIF)

S3 Fig Adjusted relative risks (95% confidence intervals) of neonatal mortality by site and SES. Relative risks are adjusted for SES category, site, site by SES interaction, maternal age, parity, formal education level, BMI category, and facility birth.
(TIF)
