# Supplementary figures and images for: The Global Network Socioeconomic Status Index as a predictor of stillbirths, perinatal mortality, and neonatal mortality in rural communities in low and lower middle income country sites of the Global Network for Women’s and Children’s Health Research
Source: PLoS One. 2022 Aug 16;17(8):e0272712. doi: 10.1371/journal.pone.0272712 (PMC9380930; doi:10.1371/journal.pone.0272712)

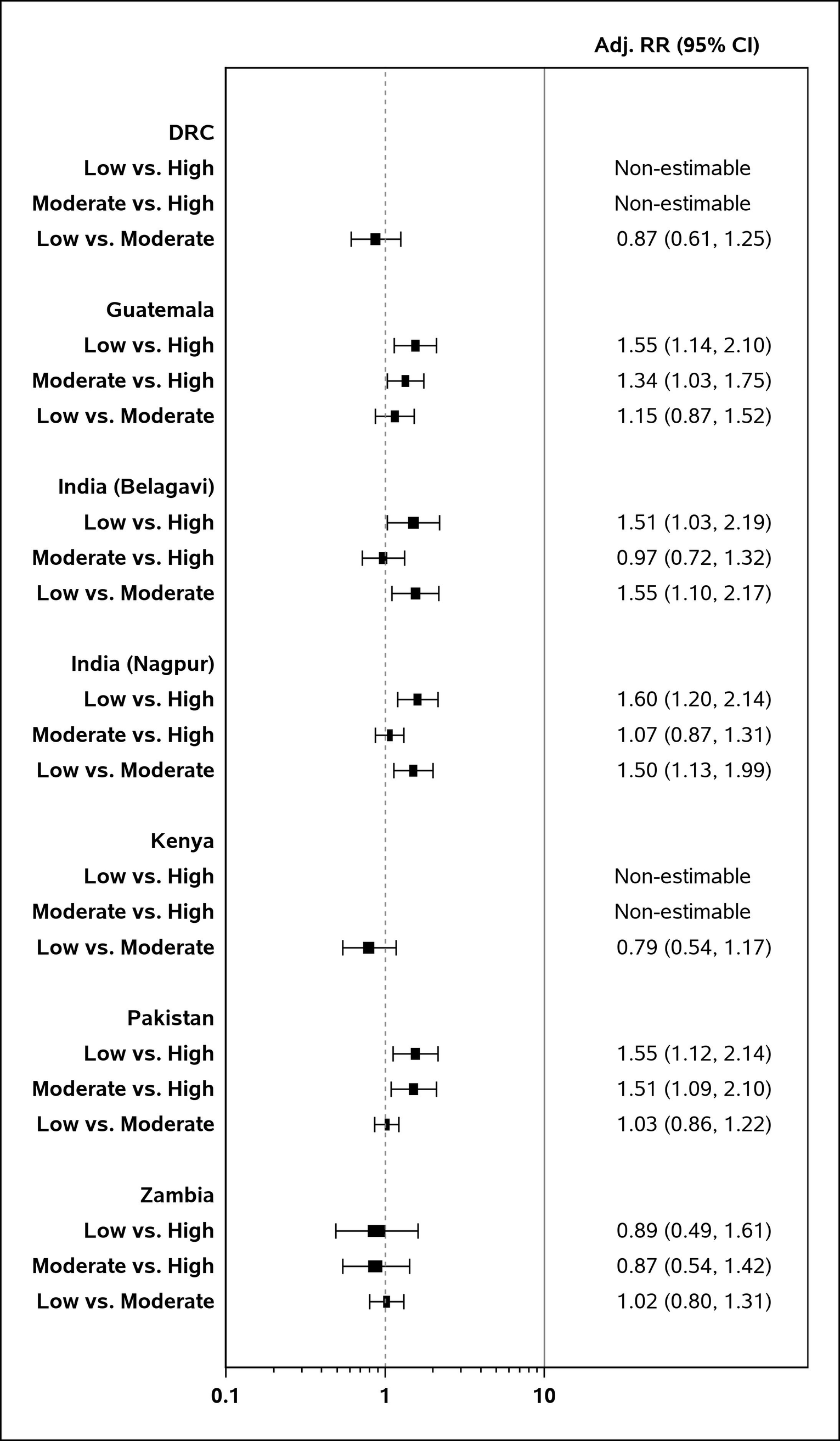

Supplement: S1 Fig — Relative risks are adjusted for SES category, site, site by SES interaction, maternal age, parity, formal education level, BMI category, and facility birth. (TIF) [file pone.0272712.s001.tif]

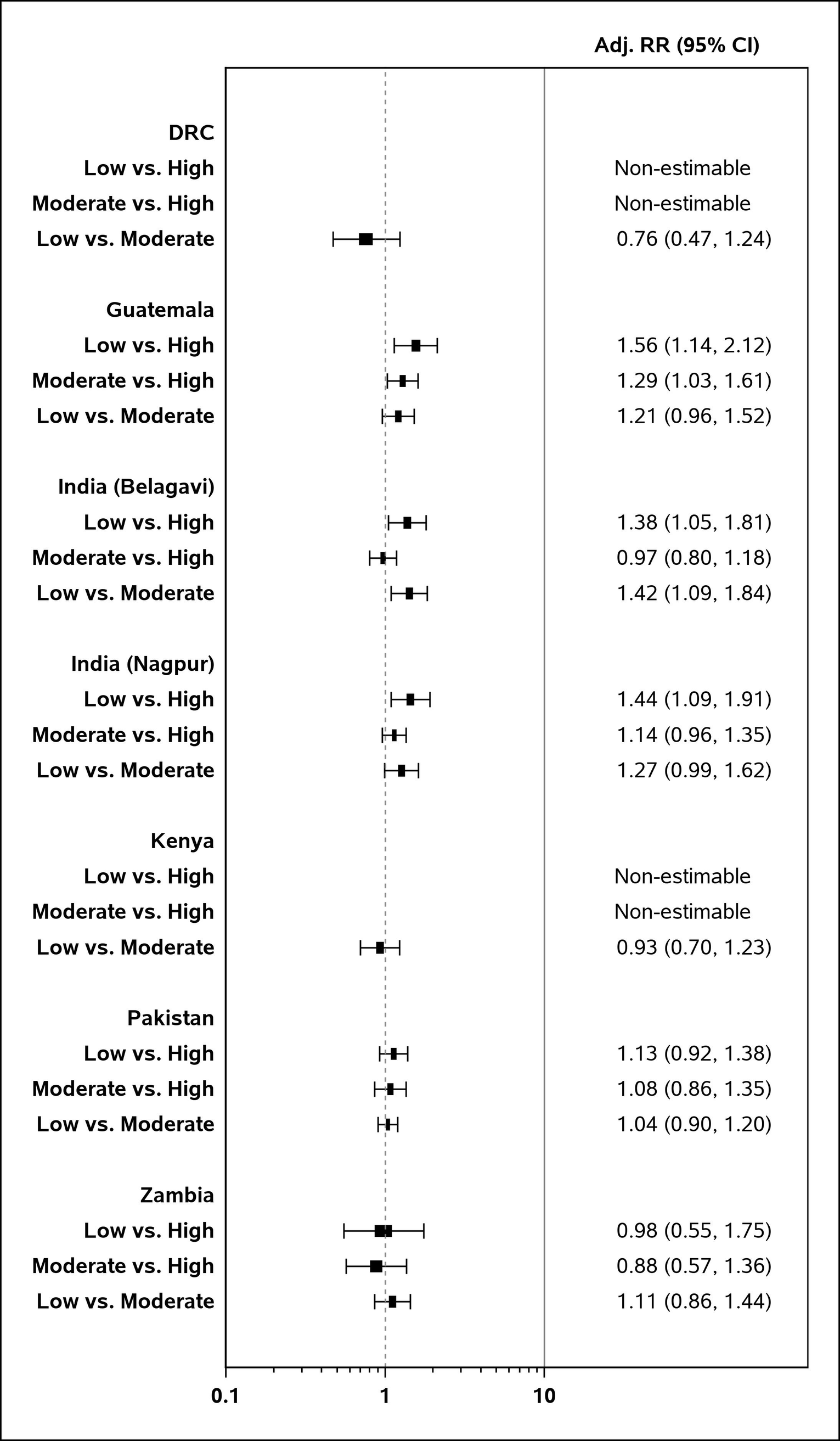

Supplement: S2 Fig — Relative risks are adjusted for SES category, site, site by SES interaction, maternal age, parity, formal education level, BMI category, and facility birth. (TIF) [file pone.0272712.s002.tif]

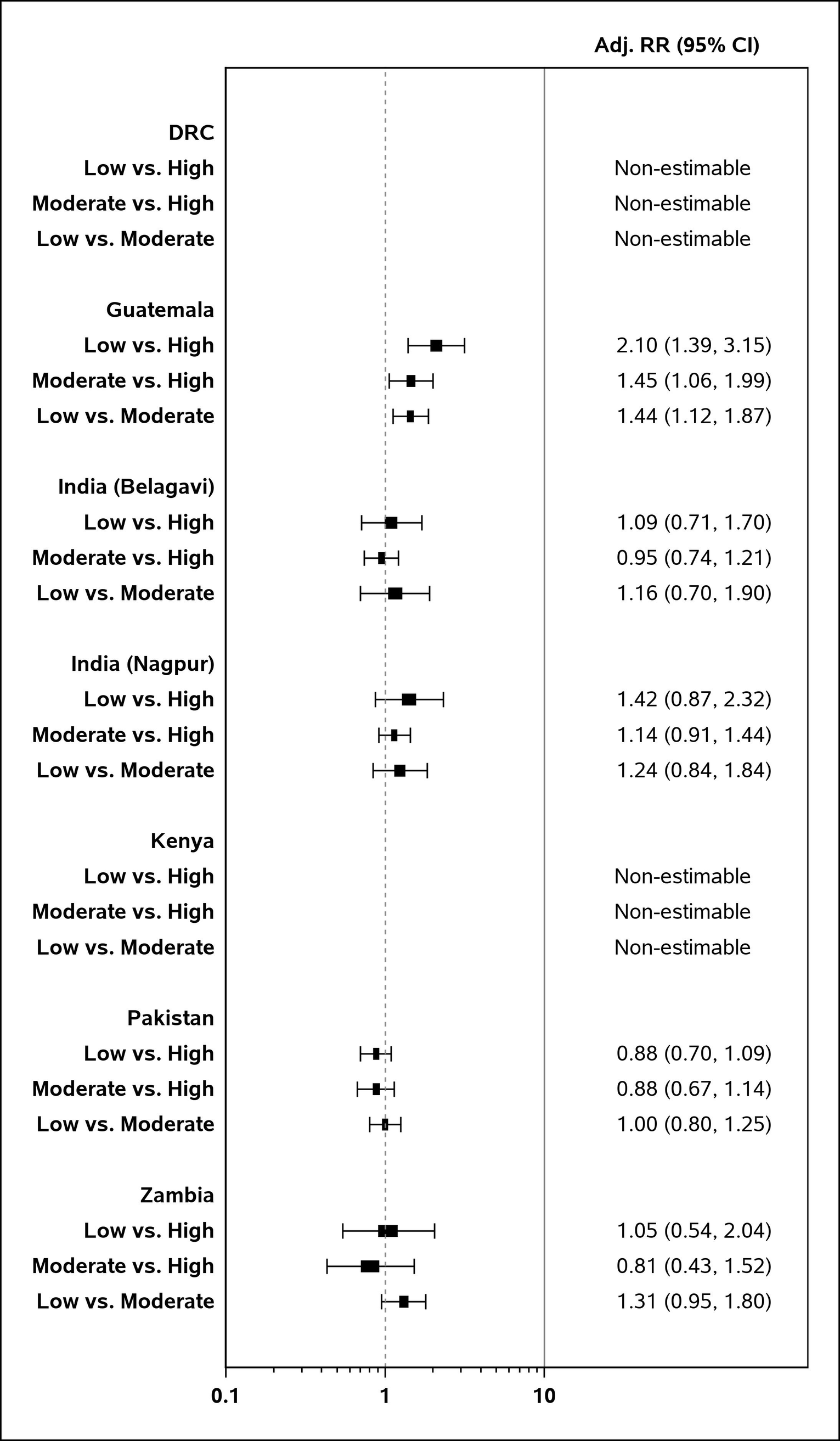

Supplement: S3 Fig — Relative risks are adjusted for SES category, site, site by SES interaction, maternal age, parity, formal education level, BMI category, and facility birth. (TIF) [file pone.0272712.s003.tif]
